# Exploring the relationship between schoolbag weight and back pain in primary school children

**DOI:** 10.1186/s13018-025-05963-1

**Published:** 2025-05-30

**Authors:** Sara Gamareldein Abdalla Khalafalla, Yousif Omer Elgaili Yousif, M. Elghazali Abuelgassim E. Mustafa, Widad Alsheikh Mostafa Alsheikh, Hiba Gamareldin A. Khalafallah, Mohammed Mubarak Mohammed Ahmed, Hozifa Mohammedd Ali Abd-Elmaged

**Affiliations:** 1 Ministry of Health, Khartoum, Sudan; 2 Alzaiem Alazhari University, Khartoum, Sudan; 3 Bahri University Faculty of Medicine, Khartoum, Sudan; 4 University of Gezira, Gezira, Sudan; 5 Orthopaedic and Traumatology, Ibrahiem Malik Teaching Hospital, Khartoum, Sudan; 6 Orthopaedics and Traumatology, Faculty of Medicine, Alzaiem Alazhari University, Khartoum, Sudan

**Keywords:** Backpain, Primary school students, School bag

## Abstract

**Background:**

Schoolbag weight in schoolchildren is a recurrent and contentious issue in education and health. Excessive schoolbag weight can lead to back pain in children, which increases the risk of chronic back pain in adulthood. This study aims to explore the pain experienced by primary school children and identify the risk factors for back pain among them.

**Materials and methods:**

This observational, descriptive, cross-sectional study was conducted among Omdurman locality in Sudan primary school students from November 1, 2020, to May 31, 2021. A multistage random sampling technique was used to select the sample. Four schools were chosen. A total of 384 students were enrolled. The data were collected using a questionnaire administered by the researcher and analyzed by SPSS version 26.

**Results:**

A total of 384 students were included in this study. A total of 192 (50%) were female, and 192 (50%) were male. The ages of the respondents ranged from 7 to 13 years, and the mean age was 11.5 ± 2. 09) SD. More than half of the studied students reported back pain 200 (52.1%); 40 (20%) were females, and 160 (80%) were males. Regarding the Visual Analogue Scale (VAS) score, 129 (64.5%) students rated their pain mild. Most 170 (85%) took medication without medical consultation. More than half of 200 (52.3%) students carried a weight greater than 15% of their body weight, and no one carried a bag with a weight less than 10%. Most students reach school by walking, which takes 10–20 min. There was a significant statistical association between the presence of back pain and older student age, male sex, carrying a bag more than 15% of one’s body weight, carrying a bag by one shoulder or side handbag, holding a bag through a morning venue and reaching school by walking for 10–20 min. P value (0.000).

**Conclusion:**

More than half of our students reported back pain 200 (52.1%); 40 (20%) were females, and 160 (80%) were males. This study highlights a strong link between the prevalence of low back pain and the lifting of heavy school bags in primary school students in the Omdurman locality. The weights of the students’ schoolbags were higher than the internationally accepted standards. Through this study we are aiming to raise awareness about the negative effects and consequences of carrying heavy schoolbags, and recommending proper scheduling of classes and providing lockers as well as transportations to decrease this phenomenon.

## Introduction

In children, nonspecific back discomfort is a common complaint that may sometimes be more frequent. Given the possible impact of recurrent nonspecific back pain that children and adolescents encounter while going about their daily lives at home and at school, educators, parents, and guardians need to be particularly aware of this issue [1, 2].

The strain on the musculoskeletal system over an extended length of time might result in musculoskeletal discomfort. In addition to the tendons and muscles, this load also affects the cervical nerves and joints, the thorax, the upper and lower back, the shoulders, the arms, and the hands. Based on epidemiological research, the prevalence rate of nonspecific back pain in children and adolescents ranges from 12 to 92%, increasing with respondent age. The following factors are frequently linked to non-specific back pain in schoolchildren: age and gender; schoolbags (heavy, improperly packed, and carried methods); poor lifestyle choices (long-term TV watching, excessive computer use, and playing video games); poor posture when sitting for extended periods of time; furniture that is not child-sized; lack of physical activity; and so on [3–5].

For schoolchildren, schoolbags are the most popular way to transport books and materials. Overweight school backpacks are an international issue that has been the subject of numerous research conducted in various nations. Back pain is the most prevalent health issue associated with large schoolbags. Heavy schoolbags can also lead to bad posture, weariness, and exhaustion, which can impair concentration and academic performance [6]. Additionally, large schoolbags can induce stress injuries, alter bone growth, and cause a body’s center of gravity to shift in the direction of the burden [7]. The body mass index (BMI) and age of the child determine which method is best to carry a schoolbag as well as the suggested weight [8].

Evaluating nonspecific back pain in school-age individuals may offer additional understanding into the potential emergence of associated diseases in later life. In order to prevent back pain’s negative repercussions, early prophylaxis is necessary [9]. Schools are vital institutions because they offer a healthy atmosphere that helps kids develop and thrive. Students most frequently bring books, supplies, and other necessities to school in bags [10]. Throughout the whole academic year, students carried their school bags five days a week. Packed lunches, sports gear, laptops, and bulky books were all within the backpacks [11]. Children’s body frames are linked to a number of musculoskeletal health issues when they carry large school bags. The weight of school bags and the unfavourable effects of carrying large loads can result in lasting damage to the spine since the musculoskeletal system of early teenage children is still developing [12].

Based on epidemiological research, the prevalence rate of nonspecific back pain in children and adolescents ranges from 12 to 92%, increasing with respondent age. Age and gender, schoolbags (heavy bags, carrying techniques, and improperly packed schoolbags), poor lifestyle choices (long-term TV watching, excessive computer use, and video game playing), poor posture when sitting for extended periods of time, mismatched furniture, inactivity, obesity, and so on are all frequently linked to nonspecific back pain in schoolchildren [13]. While lower back pain (LBP) is not a fatal illness, it is a major contributor to lost productivity and job absenteeism. It significantly affects life quality [14]. According to epidemiological research, school-age children experience a higher than anticipated prevalence of back pain, with a regional prevalence of 74% [15].

We observed an alarming increase in pain among schoolchildren, raising red flags. The development of back pain in children is of concern since it increases the risk of developing chronic back pain in adulthood. Knowledge concerning LBP among children might provide insight into its risk factors and potential underlying causes in Sudan. To our knowledge, no published studies have measured the prevalence of LBP and associated back bag weight as risk factors among Sudanese school children within the past few years. Moreover, this study may be helpful for pediatricians and relevant medical professionals to be more critical in investigating the relevant risk factors among this group of patients and for better selection of overall management strategies for better short-/long-term outcomes in Sudan. The study aims to raise the awareness of critical effects of heavy schoolbags among parents, guardians, head masters and school teachers as well to decrease this phenomenon and its consequences.

## Materials and methods

This was an observational descriptive cross-sectional community-based study. The study was conducted in 4 governmental primary schools. The study population included primary school students of both sexes aged 7 to 14 years who lived in the Omdurman locality and were studying at primary governmental schools. They were generally healthy students whose parents agreed to participate in this study. Individuals who were unable to stand on the weighing scale, unable to carry school bags, or who had back deformities were not included in this study. A total random sample of 384 primary school children was used, including 192 boys and 192 girls. A multistage random sampling technique was used to select the sample. First, Omdurman city was chosen randomly from 3 cities in Khartoum state, Sudan. Four primary governmental schools (2 for males and 2 for females) were selected using a stratified random technique from a list of primary schools obtained through the Directorate of Education in the Omdurman locality. Then, students inside the school were selected via simple random techniques from a list of primary school students obtained from the headmaster of the school.

The sample size was calculated using the online software Epi-info according to the following equation:

$$\:\frac{{z\:}^{2}X\:p\:(1-p)}{{e}^{2}}$$

Sample size =ــــــــــــــــــــــــــــــ.

$$1 + \;\left( {{{{z^2}Xp(1 - p)} \over {{e^2}N}}} \right)$$

N = population size (89042 students) according to the Omdurman locality, e = margin of error (5%), z = z - score standard deviation (1.96), p = expected proportion in population based on pilot study (50%). After reviewing the related literature, the researchers developed a two-part structural questionnaire sheet. The first part collected the sociodemographic data of the students, such as age, sex, grade, student weight, and school bag weight. The second part collected information about school bags using close-ended questions about school bags, such as their type, method, and frequency of carrying, and respondents’ opinions of the weight of their school bags. A scale with weight detectors was used to measure the student’s body weight and the weight of the school bag. Self-reports (numeric pain rating scale) were used to assess the intensity of pain experienced by the students. The questionnaire was in Arabic language.

Five experts tested the content validity of the tools in the field. A pilot study was conducted on 10 students to evaluate the tools’ applicability and clarity and estimate the time needed to complete the questionnaire; participants were excluded from the total sample. The researcher completed the questionnaire by conducting direct interviews with the students during the activity hours. Each class was separated, and the students were asked to answer questions individually after providing a detailed explanation of the study objectives and procedure as well as statements that reassured them that the information was confident and used only for the research. The use of the numeric pain rating scale (VAS) was explained. The students were asked to choose the appropriate number, which was matched to the pain intensity if the number was zero on the scale, indicating “no pain,” 10, which indicated “worst possible pain”; a score ranging from 2 to 3 indicated mild pain, a score ranging from 4 to 6 indicated moderate pain and a score more excellent than 7 indicated severe pain.

The weighing scale was flat in a corner of the classroom and set to zero. The student was asked to remove his shoes and weigh himself when carrying the school bag first and then without the bag for the second time; the difference between the two measures was the bag’s weight. The school bag weight was considered appropriate if it ranged from 10 to 15% of the student’s body weight while the heavy load exceeded 15%.

The data were entered, cleaned, and analyzed using SPSS version 26.0. The descriptive statistics are presented as frequency tables with percentages and graphs. Means and standard deviations are presented with relevant graphical representations for quantitative data. Bivariable analysis was performed to determine the associations between the different risk factor variables and the other relevant demographic/clinical characteristics with chi-square tests (for categorical variables) and t-tests (for quantitative variables). A P value of 0.05 or less was considered to indicate statistical significance. The data were analyzed using univariable tables, cross-tabulations (bivariable tables), figures, and narrative illustrations.

Written ethical clearance and approval for conducting this research were obtained from the Sudan Medical Specialization Board Ethical Committee with the ID SMSBE22000128. Written permission was obtained from the administrative authority of all governmental schools under study. Study data/information were used for research purposes only. Privacy issues were intentionally considered. Participation is voluntary. Any participant has his/her right to withdraw at any stage. Written consent was obtained from the parents and students after they were fully informed about the study details. The work has been reported in line with the STROCSS criteria [16].

## Results

The study investigated the entire proposed sample. Three hundred eighty-four primary school students with no previous health problems were enrolled in this study. All the students studied in the 1st floor class. One hundred ninety-two (50%) of the studied pupils were females, and 192 (50%) of them were males. The M: F ratio was 1:1.

The ages of the responders ranged from 7 to 13 years. The mean age was (11.5) + or - (2.09) SD years, as shown in Table 1.

**Table 1:** Age and gender groups of the study population

Age group	Frequency	Percent
7–8 years	55	14.3%
9–10 years	104	27.1%
11–12 years	105	27.3%
13–14 years	120	31.3%
**Gender**		
Female	192	50%
Male	192	50%
**Total**	**384**	**100.0%**

**Fig. 1:**
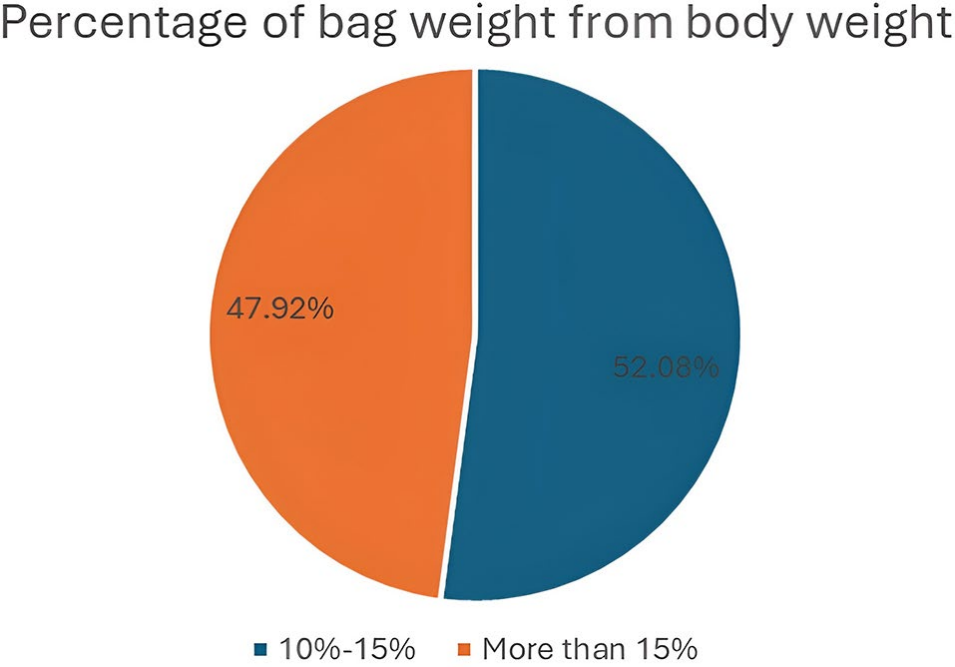
Percentage of bag weight relative to the body weight of participants

The weights of the students’ and school bags were measured to calculate the percentage of students’ bags relative to their weight. The study revealed that 200 (52.1%) of the students carried bags with weights more significantly than 15%, and 184 (47.9%) carried bags with weights between 10% and 15%. No one carried bags with weights less than 10% of their weight, as shown in Fig. 1.

Regarding how the studied students held their school bags, 190 (49.5%) held them with one shoulder, 184 (47.9%) held them with both shoulders, and only 10 (2.6%) held them with their side hands, as shown in Table 2.

**Table 2:** Methods of holding school bags among the study population

Method of holding a bag	Frequency	Percent
By one shoulder	190	49.5%
Both shoulder	184	47.9%
Side hand	10	2.6%
**Total**	**384**	**100.0%**

**Fig. 2:**
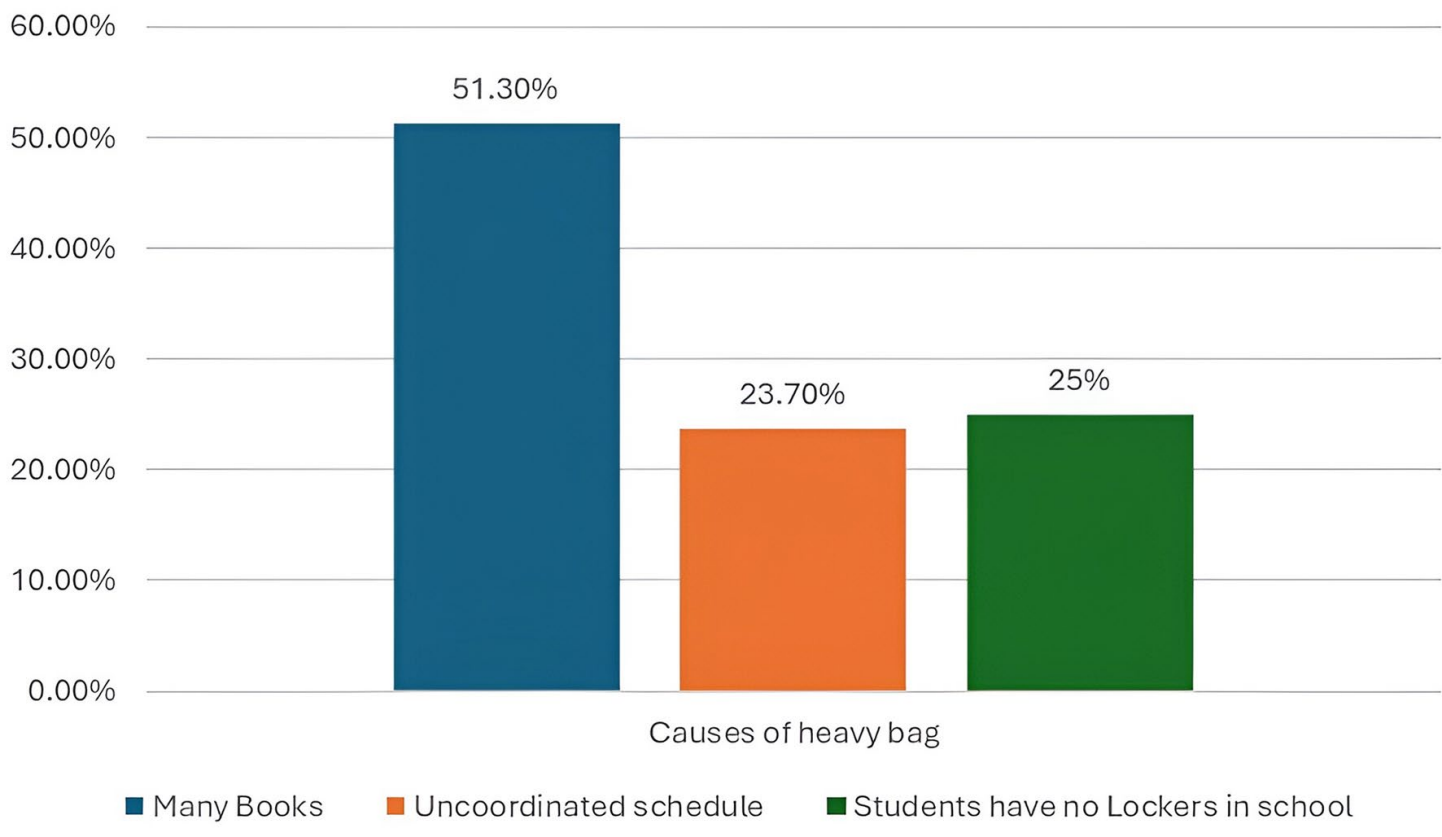
shows the causes of heavy bags as reported by participants

Regarding the causes of heavy school bag weight according to the responses of the students, 197 (51.3%) of the students declared that the cause was too many books, 96 (25%) stated that they had no lockers and 91 (23.7%) of them said uncoordinated schedules, as shown in Fig. 2.

Regarding students’ reactions to heavy school bags, 147 (38.3%) stated that they swayed left, right, or bent forward, 136 (35.4%) mentioned that their parents helped them carry school bags, and 101 (26.3%) students responded that they took a rest while they were carrying school bags, as shown in Table 3.

**Table 3:** Reactions of the students to heavy school bags

Reaction of students to heavy bag	Frequency	Percent
Sway left, right or bend forward	147	38.3%
Parents help in carrying school bag	136	35.4%
Take a rest while carrying the school bag	101	26.3%
**Total**	**384**	**100.0%**

Nearly half of the students (52.1%) said they carried bags through the morning queue, and 47.9% did not.

Regarding the transportation methods used by the students, 200 (52.1%) were walking to school, 144 (37.5%) reached school by bus, and the remaining 40 (10.4%) were by private car. Regarding the time spent reaching their schools, 267 (69.5%) students took 10–20 min to reach their schools, 75 (19.5%) took more than 20 min to reach their schools, and only 42 (11%) took less than 10 min to reach their schools.

**Fig. 3:**
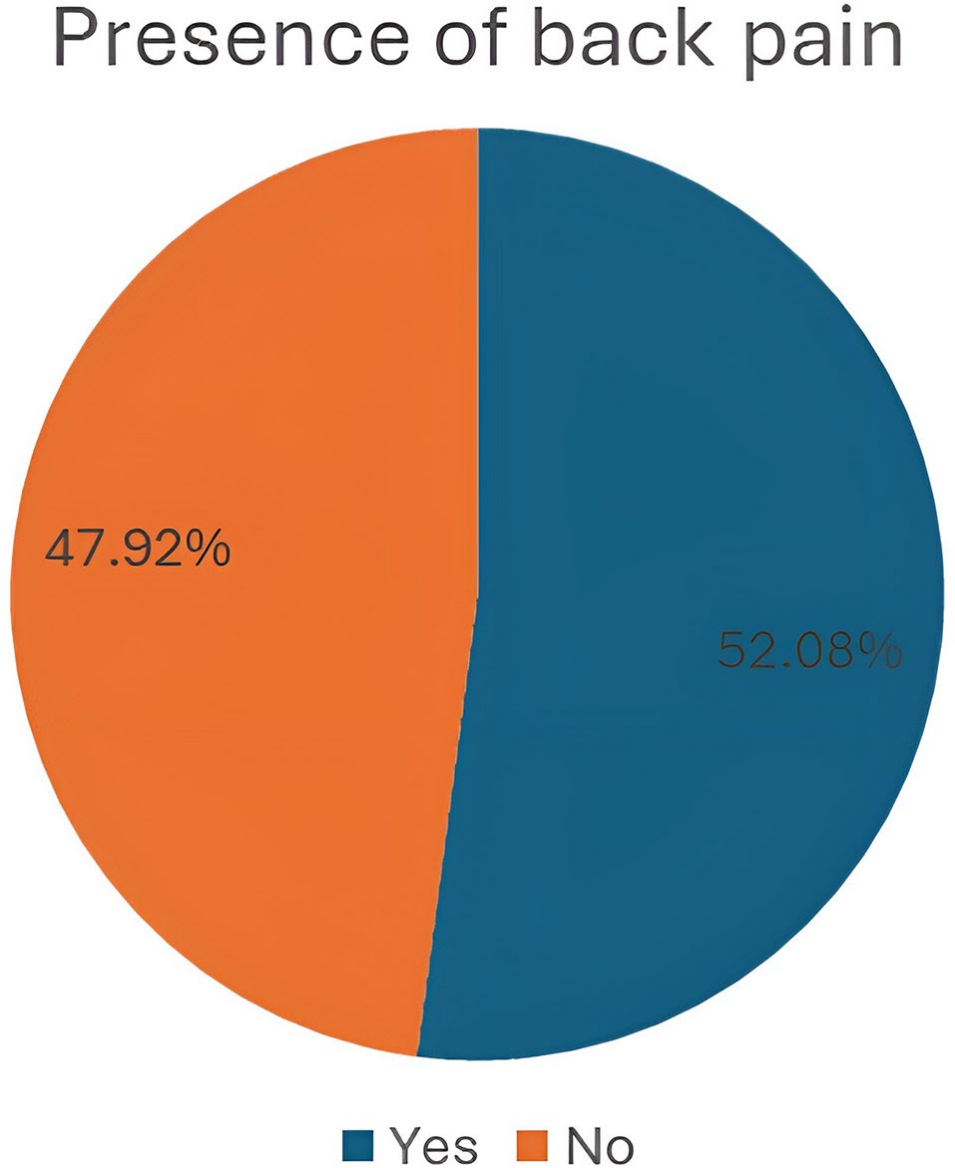
The presence of back pain among the studied students

The current study reported that more than half of the students had back pain 200 (52.1%)0.40 (20%) from them were females, and 160 (80%) were males. And one hundred eighty-four (47.9%) students reported no back pain., as shown in Fig. 3.

Regarding the Visual Analogue Scale (VAS) score, 129 (64.5%) of the students rated their pain as mild, 66 (33%) of the students rated it as moderate, and only 5 (2.5%) of the students rated it as severe, as shown in Table 4.

**Table 4:** The intensity of back pain according to the VAS score for the students who experienced back pain

Intensity of pain on VAS	Frequency	Percent
0 = No pain	0	0
1–3 = mild pain	129	64.5
4–6 = moderate pain	66	33
7–10 = sever pain	5	2.5
**Total**	**200**	**100.0**

Regarding the effect of pain on the student’s daily life, 66 (33%) of them had sleep disturbances from pain, 55 (27.5%%) could not walk straight, and 79 (39.5%) of the students stated that they had pain all day, as shown in Table 5.

**Table 5:** The effects of pain on the daily life of the study population who experienced back pain

Effect of pain	Frequency	Percent
No effect	0	0
Can’t get straight	55	27.5
Pain all day	79	39.5
Sleep disturbance	66	33
**Total**	**200**	**100.0**

The students’ reaction to pain was that 170 (85%) of them took medications without medical request, but 30 (15%) of them went to the physician, as shown in Table 6.

**Table 6:** The students’ reactions to pain

Reaction of student to pain	Frequency	Percent
Go to physician	30	15
Take medications without medical request (analgesia)	170	85
Have no reaction	0	0
**Total**	**200**	**100.0**

Bivariate analysis of back pain and student sociodemographic characteristics revealed a significant association between the presence of back pain and older students aged 13–14 (P value 0.000), as shown in Table 7.

**Table 7:** Back pain in relation to participant age

Age	Presence of pain		Total	P value
	Yes	No		
7–8 years	15 (3.9%)	40 (10.4%)	55 (14.3%)	0.000
9–10 years	50 (13.0%)	54 (14.1%)	104 (27.1%)	
11–12 years	55 (14.3%)	50 (13.0%)	105 (27.3)	
13–14 years	80 (20.8%)	40 (10.4%)	120 (31.3)	
Total	200 (52.1%)	184 (47.9%)	384 (100%)	

Additionally, there was a significant association between the presence of back pain and male sex (P value 0.000), as shown in Table 8.

**Table 8:** Association between back pain and sex

Gender	Presence of pain		Total	P value
	Yes	No		
Male	160 (41.7%)	32 (8.3%)	192 (50%)	0.000
Female	40 (10.4%)	152 (39.6%)	192 (50%)	
Total	200 (52.1%)	184 (47.9)	384 (100%)	

The study showed that back pain was more prevalent among students who carried school bags for more than 15% of their body weight (P value 0.000).

Additionally, there was a significant statistical association between the presence of back pain and the use of a holding bag, which was significantly more strongly associated with the use of a carrying bag with one shoulder or side hand than with the use of a carrying bag with both shoulders (P value 0.000), as shown in Table 9.

**Table 9:** Relationships between bag pain and bag holding methods reported by participants

Back holding	Presence of pain		Total	P value
	Yes	No		
One shoulder	190 (49.5%)	0	190	0.000
Two shoulders	0	184 (47.9%)	184	
Side hand	10 (2.6%)	0	10	
Total	200 (52.1%)	184 (47.9)	384 (100%)	

There was an insignificant statistical association between back pain, the causes of heavy bags, and students’ reactions to heavy bags (P value 0.248).

Additionally, there was a significant association between back pain and school bag use throughout the morning queue (P value 0.000).

As shown in Tables 10 and 11, there was a significant association between the presence of back pain and the time required to reach the school (P value 0.000).

**Table 10:** Back pain in relation to the transportation method used to reach the school by participants

Transportation method used to reach the school	Presence of pain		Total	P. v
	Yes	No		
Walking	200 (52.1%)	0 (0.0%)	200 (52.1%)	0.000
Special car	0 (0%)	40 (10.4%)	40 (10.4%)	
The bus	0 (0%)	144 (37.5%)	144 (37.5%)	
Total	200 (52.1%)	184 (47.9%)	384 (100%)	

**Table 11:** Back pain in relation to the time needed to reach the school

Time needs to reach the school	Presence of pain		Total	P. v
	Yes	No		
Less than 10 min	0 (0.0%)	42 (10.9%)	42 (10.9%)	0.000
10–20 min	200 (52.1%)	67 (17.4%)	267 (69.5%)	
More than 20 min	0.0%	75 (19.5%)	75 (19.5)	
Total	200 (52.1%)	184 (47.9%)	384 (100%)	

This study revealed significant associations between the presence of back pain and older student age, male sex, carrying a bag more than 15% of one’s body weight, carrying one shoulder or side handbag, holding one shoulder or side handbag through a morning queue, and reaching school by walking for 10–20 min.

## Discussion

Heavy school bags are a global problem, and several studies have addressed this problem in different countries. Musculoskeletal pain is a condition caused by the load of the musculoskeletal system over a more extended period. The most common health problem related to heavy school bags is back pain.

Several reviews have determined that schoolchildren’s most common cause of nonspecific back pain is schoolbag use and related factors. Some studies have demonstrated that heavy schoolbags and the wrong method of carrying heavy schoolbags during this critical period of life have adverse effects on the growth of the musculoskeletal system, resulting in back pain that has increased over time, predisposing children to mobility problems in adulthood [14].

The present study revealed that more than half (52.1%) of school-aged children suffered from back pain, which aligns with a study that reported a 55% prevalence of back pain [17].

Another study reported that approximately three-fourths of school children in the studied sample had suffered from back pain in the month prior to the study, which is greater than that reported in our study [4]. Moreover, our findings were lower than those of a study that revealed that the prevalence of back pain in children and youth aged 10–19 years living in southeastern Poland was 76% [15]. We reported a greater prevalence of back pain among primary school students than in other studies performed in Uganda and Saudi Arabia, which were 37.8% and 42%, respectively [18, 19]. This difference in the prevalence of back pain may be due to differences in age group, sex, sample size, and the children’s social environment.

Regarding gender, the present results showed that male students were four times more likely to suffer from back pain than female students. There was a significant statistical association between male sex and the occurrence of back pain among primary school students (P value = 0.000). This result was inconsistent with several studies showing that gender is a significant factor in the development of back pain among school children. Girls were more likely to report back pain than boys of the same age, which is in contrast to a study reporting that female sex is a predictor of back pain [4]. This may be related to physiological differences between the two sexes. Additionally, another study reported that the occurrence of back pain due to school bags was related to sex, which is inconsistent with our study, as it is common in males [20]. In Spain, a study concluded that girls presented a greater risk of back pain than boys did, in contrast to our study, in which back pain was joint in males [21].

Most of our respondents reported that the intensity of their back pain, according to the VAS score, was mild (64%), and only 2.5% of the students experienced severe back pain, which is consistent with a previous study reporting that mild pain is prevalent [15]. Another study reported that moderate pain is prevalent in their study, as more than half of responders stated that their pain is moderate, in contrast to our study, in which mild pain was expected [4].

Regarding impairment in daily activities, the results showed that all schoolchildren who suffered from pain reported that the pain prevented them from performing daily activities such as studying, participating in leisure activities, playing, and disturbing their sleep. However, another study revealed that one-fourth of students’ daily activities were affected [4].

Regarding students’ reactions to back pain, the present study revealed that approximately 70% of school children who experienced pain reported taking analgesics without doctor consultation, and only one-third sought medical advice. In contrast, in another study, approximately 14% of patients alleviated pain with analgesics without doctor consultation, and only 10% of patients used analgesics prescribed by doctors [4].

This study revealed a significant association between the presence of back pain and age (13–14 years, P value = 0.000); in contrast, another study revealed that back pain was not significantly associated with the age of children or adolescents [22]. In contrast, other studies have shown a significant association between age and the frequency of occurrence of back pain, with a significant increase in prevalence as age increases, which agrees with our study [15, 23]. The age group (13–14) included participants who had completed their last and most challenging work in two primary school classes, who had multiple books available for study, and who had longer stays at school, all of which may have contributed to the development of back pain and the cumulative presence of a bag for the previous study years. This age group seems to have impacted the development of their back pain.

Concerning school bag weight as a percentage of body weight (% of BW), the present study showed that 52.3% of students carried a bag with a weight greater than 15% of their body weight, 184 (47.9%) of students carried a bag with a weight between 10% and 15% of their body weight, and no one who carried a bag with a weight less than 10% of their weight. Internationally accepted standard weights for students’ schoolbags should not exceed 10–15% of their body weight [19]. There was a significant statistical association between the presence of back pain and the presence of a bag with a weight greater than 15% of their body weight, P value (0.000). This finding is consistent with that of another study [4]. Additionally, a study reported that 72.46% of studied school children carried bags for more than 15% of their body weight, which is greater than that reported in our study [19]. In contrast, another study conducted among students revealed that 7.1% of the studied school children carried bags that weighed more than 15% of their body weight. This difference may be related to sociodemographic and cultural factors [22]. In contrast to our study, another study demonstrated no associations between back pain and the weight of school bags or between the weight of school bags and the percentage of body weight [24].

Our study revealed that carrying school bags on one shoulder or one hand increases the risk of back pain compared with carrying school bags on both shoulders (P value = 0.000). This finding was to the findings from review studies that showed that carrying schoolbags on one shoulder or by one hand causes asymmetry in muscle activity, encourages lateral spinal bending, and might lead to changes in shoulder level and the development of shoulder, neck, and lower back pain even if the bag weight constitutes 10% of the child’s weight [25]. One study reported a significant association between low back pain and bag carriage, consistent with ours [18]. In contrast, another study revealed no significant relationship between the method of carrying school bags and the prevalence of back pain [24].

Concerning the methods and times used to reach school, in the present study, we found a significant statistical association between the presence of back pain and the use of walking as a transportation tool to school and the use of 10–20 min of walking to reach school PV (0.000). This finding aligns with another study, but he reported more than 20 min [26].

Our study revealed significant associations between the presence of back pain and older student age, male sex, the ability to carry more than 15% of one’s body weight, the ability to carry one shoulder or side handbag, the ability to hold one shoulder or side handbag through a morning venue, and the ability to reach school by walking for 10–20 min.

## Conclusion

Most of the students reported having a backache. It commonly affects male students. The majority of the students in our study carried a schoolbag of more than 15% of their body weight. The weight of the students’ schoolbags was higher than the international standards recommended by the WHO. There was a significant statistical association between the presence of back pain and older student age, male sex, carrying a bag by one shoulder or side handbag, holding a bag through a morning venue, and reaching school by walking for 10–20 min. This study highlights a strong link between the prevalence of low back pain and the lifting of heavy school bags in primary school students.

As we reflect through this cross-sectional study the adverse effects of carrying heavy schoolbags, we aim to raise awareness about it, emphasize the importance and necessity of decreasing the weight of schoolbags through appropriate scheduling of classes, providing lockers for students, and offering proper transportation for those students who used to walk to school.

The study limitations included that this study was observational cross-sectional study that limits the casual inference between school bag characteristics and back pain. The study was localized to governmental primary schools in Omdurman locality, which may decrease the generalizability of the findings to private schools or other regions. Pain assessment relied on student self-report, which may introduce recall bias or subjective variability. Finally, despite the use of validated tools and expert review, the reliance on interview-based data collection may have introduced interviewer bias.

## Data Availability

Data cannot be shared openly but are available on request from authors.
